# Mitochondria Metabolism Regulates Glucose–Lipid Homeostasis in Neurodegenerative Diseases

**DOI:** 10.34133/research.0912

**Published:** 2025-10-02

**Authors:** Jianliang Huang, Can Zhang, Cheng Huang, Kun Deng, Yun Xiao, Wei Gao, Minghua Wu, Mingsheng Lei

**Affiliations:** ^1^ Zhangjiajie Hospital Affiliated to Hunan Normal University, Zhangjiajie 427000, Hunan, China.; ^2^ The Key Laboratory of Carcinogenesis of the Chinese Ministry of Health, The Key Laboratory of Carcinogenesis and Cancer Invasion of the Chinese Ministry of Education, Cancer Research Institute, School of Basic Medicine, Central South University, Changsha 410000, Hunan, China.; ^3^ Changsha Central Hospital, Changsha 410000, Hunan, China.; ^4^ Furong Laboratory, Central South University, Changsha 410000, Hunan, China.; ^5^ Zhangjiajie College, Zhangjiajie 427000, Hunan, China.

## Abstract

Neurodegenerative diseases represent a major health threat, with dysfunction in energy metabolism and imbalance in glucose–lipid homeostasis constituting key pathogenic factors. As the cell’s energy hub, mitochondria are closely associated with neurodegenerative diseases, such as Alzheimer’s and Parkinson’s diseases. However, the precise mechanism by which mitochondrial energy metabolism affects glucose–lipid homeostasis remains unclear. This review summarizes currents insights into the role of mitochondrial function in energy metabolism and glucose–lipid regulation in neurodegenerative diseases. We examined how mitochondrial dynamics, oxidative phosphorylation, calcium homeostasis, and key signaling pathways—AMP-activated protein kinase/mammalian target of rapamycin, peroxisome proliferator-activated receptor gamma coactivator 1-alpha, and Sirtuin 1—contribute to neuronal energy balance and metabolic regulation. We further explored the impact of other organelles on mitochondria and how the dynamic switching of mitochondrial morphology and function disrupts the critical glucose–lipid homeostasis. By focusing on mitochondrial dysfunction, metabolic disorders, and their interactions, we introduce the mitochondria-centered multi-organelle–energy metabolic–glucose–lipid homeostasis (MMH) network as a unifying theoretical framework that positions the progressive loss of metabolic flexibility as the fundamental essence of neurodegenerative disorders. The MMH network furnishes a novel lens through which the shared mechanistic underpinnings of neurodegenerative diseases can be deciphered, and thereby enable earlier diagnosis and precision therapeutics.

## Introduction

Neurodegenerative diseases (NDDs) are a growing global public health concern, characterized by selective neuronal degeneration and irreversible synaptic damage, leading to progressive cognitive decline or motor impairment [1]. Alzheimer’s disease (AD), Parkinson’s disease (PD), and Huntington’s disease (HD) are prominent examples, contributing importantly to disability in older adults [2,3]. The median survival time for certain diseases, such as amyotrophic lateral sclerosis, is only 5 to 15 years, with a 5-year survival rate of less than 40% [4]. Current treatments—such as cholinesterase inhibitors (e.g., donepezil) and glutamatergic modulators (e.g., memantine), often combined with cognitive-behavioral therapy—offer only temporary symptom relief and do not target the underlying pathology [5]. Disease progression is influenced by factors including age, genetic mutations (e.g., *APOE-ε4* and *PARK* genes), environmental exposures, and metabolic disturbances [6]. Among these, alterations in glucose metabolism and mitochondrial DNA (mtDNA) are critical to NDDs. Studies have shown that glucose metabolism in the temporal cortex of patients with AD is significantly reduced, which is associated with Aβ deposition [7]. Furthermore, a high proportion of mtDNA deletions has been observed in the substantia nigra of patients with PD, which indicates that mitochondrial metabolic disturbances are central to the pathology of NDDs [8]. Neurons depend on mitochondria for energy and functions, such as development, synaptic plasticity, calcium regulation, and apoptosis [9].

Mitochondria occupy a considerable volume proportion in neurons, which may vary depending on the type of neuron. This highlights the critical role of mitochondria in neurons [10]. As metabolic hubs, mitochondria regulate glucose metabolism via oxidative phosphorylation (OXPHOS) and the tricarboxylic acid (TCA) cycle and balance lipid metabolism through β-oxidation [11]. They control neuronal energy supply, demand, and reprogramming via signaling pathways, such as AMP-activated protein kinase (AMPK)/mammalian target of rapamycin (mTOR) and peroxisome proliferator-activated receptor gamma coactivator 1-alpha (PGC-1α), maintaining metabolic homeostasis [12]. In NDDs, mitochondrial dysfunction and metabolic disorders are mutually causal: impaired mitochondrial respiratory chain, decreased adenosine triphosphate (ATP) production, oxidative stress, and calcium imbalance disrupt glucose and lipid metabolism, whereas metabolic disorders, in turn, exacerbate mitochondrial damage, and thereby create a vicious cycle of accelerated degeneration [13,14]. The “mitochondrial dysfunction–energy metabolism disorder–glucose–lipid imbalance” cascade is a key pathological process in NDDs [15].

Recent studies have revealed that mitochondria do not function independently; rather, they form specialized membrane contact sites with various membrane-bound organelles, such as the endoplasmic reticulum, known as mitochondria-associated membranes (MAMs), to create a highly coordinated intracellular interaction network. This network profoundly impacts the dynamic balance of glucose and lipids within neurons by regulating mitochondrial metabolic states, serving as a crucial foundation for maintaining neuronal metabolic homeostasis [16]. Based on this understanding, in recent years, research focus has gradually shifted from viewing mitochondrial dysfunction in isolation to understanding its dynamic regulation within complex cellular networks. However, the current state of symptomatic treatment reflects a limited understanding of mitochondrial energy imbalance in NDDs, particularly regarding the influence of other organelles on mitochondria and how the dynamic switching of mitochondrial morphology and function disrupts critical glucose–lipid homeostasis. This underscores the necessity for in-depth studies on the mitochondria-centered multi-organelle–energy metabolic–glucose–lipid homeostasis (MMH) network. Breakthroughs in high-throughput molecular analysis techniques, such as single-cell sequencing, real-time metabolic flux analysis, super-resolution microscopy, and real-time monitoring of mitochondrial function using fluorescent probes, have improved the precision of analyzing mitochondrial morphology, function, and their interactions with other organelles [17,18]. These technological advancements provide a solid experimental foundation for validating and deepening the MMH network theory, and thereby facilitate the systematic understanding of the pathogenesis of NDDs at the molecular, cellular, tissue, and even individual levels. This review systematically analyzes the regulation of energy metabolism by mitochondria and its disruption of glucose–lipid homeostasis to elucidate how alterations in mitochondrial function reshape neuronal metabolic patterns.

**Fig. 1.**
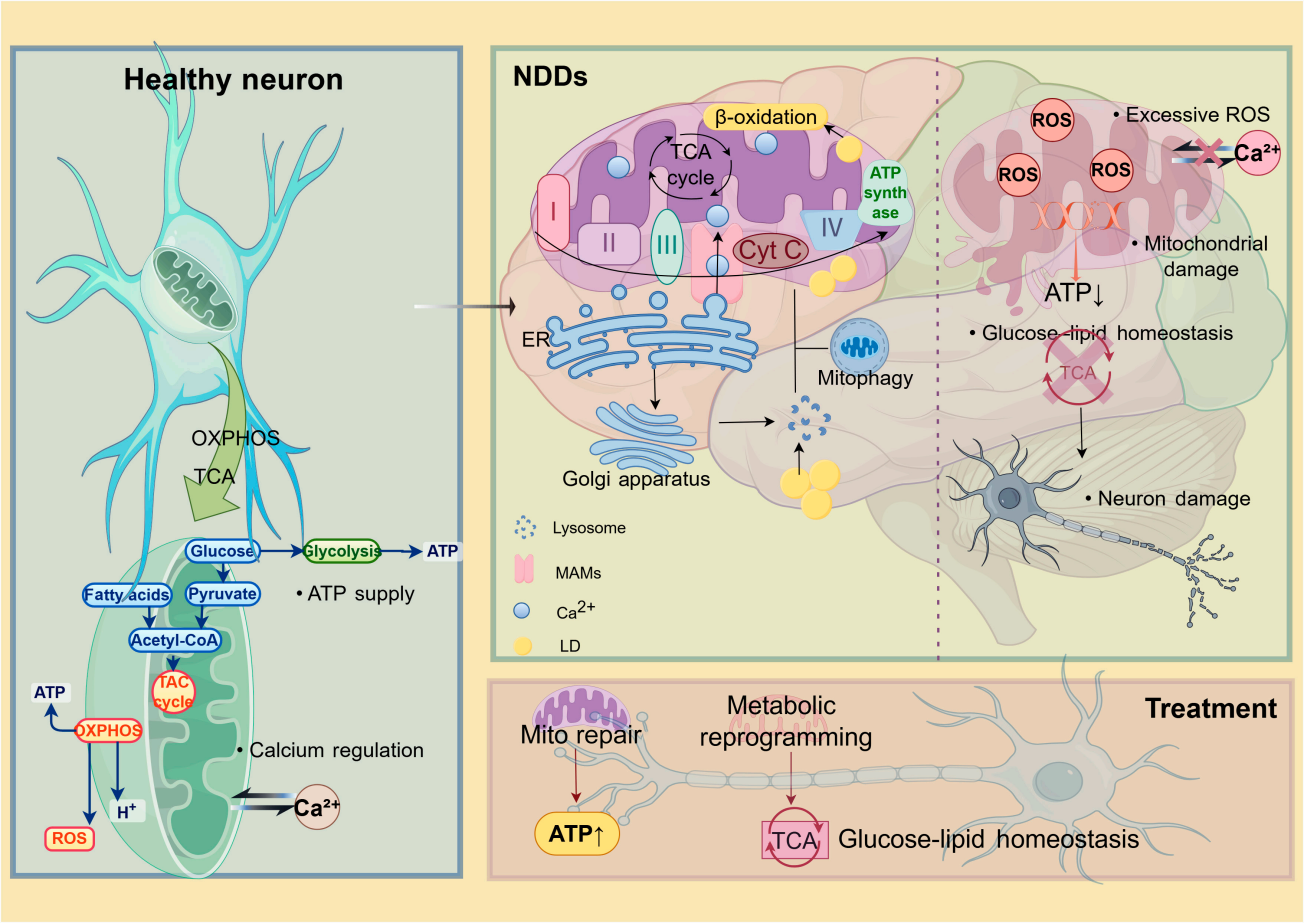
The mitochondria-centered multi-organelle–energy metabolic–glucose–lipid homeostasis network. Healthy neurons (left) maintain ATP supply, calcium homeostasis regulation, and glucose–lipid metabolic balance through normal OXPHOS and the TCA. Mitochondrial dysfunction leads to excessive ROS production, reduced ATP generation, and calcium homeostasis imbalance, which triggers metabolic disorders. This dysfunction forms a vicious cycle with glucose–lipid metabolic imbalance that ultimately results in NDDs (upper right), characterized by mitochondrial damage, disruption of glucose–lipid homeostasis, and neuronal degeneration. Simultaneously, organelles such as the Golgi apparatus, lysosomes, and endoplasmic reticulum exacerbate mitochondrial metabolic disorders by forming MAMs or producing LD. Based on the MMH network theory, a systematic targeted therapeutic strategy is proposed (lower right): restoring ATP generation through mitochondrial repair and reconstructing the TCA cycle and glucose–lipid balance via metabolic reprogramming, and thereby interrupting the pathological cascade of “mitochondrial dysfunction–energy metabolism disorder–glucose–lipid imbalance”.

We explore the MMH network, the regulatory mechanisms of multi-organelle interactions, and the relationship between the dynamic changes in mitochondrial morphology and function, while focusing on its role in maintaining neuronal glucose–lipid homeostasis and its potential as a therapeutic target. Our goal is to provide a theoretical foundation for future strategies aimed at restoring neuronal glucose–lipid homeostasis to prevent and treat NDDs (Fig. 1).

## Mitochondrial Structure and Metabolic Regulatory Interactions

### Mitochondrial structure and quality control

Mitochondria serve as the primary energy-generating organelles in cells, with specialized structures essential to their function. Defined by 2 membranes, the outer membrane forms a sheath whereas the inner membrane folds into cristae that create a cavity and increase the surface area [19]. This expanded surface supports the electron transport chain and ATP synthase. The integrity of cristae is critical for assembling respiratory complexes and ensuring efficient electron transport. Moreover, the inner membrane also encloses the mitochondrial matrix, which contains key components, such as TCA cycle enzymes, mtDNA, and ribosomes [20].

**Fig. 2.**
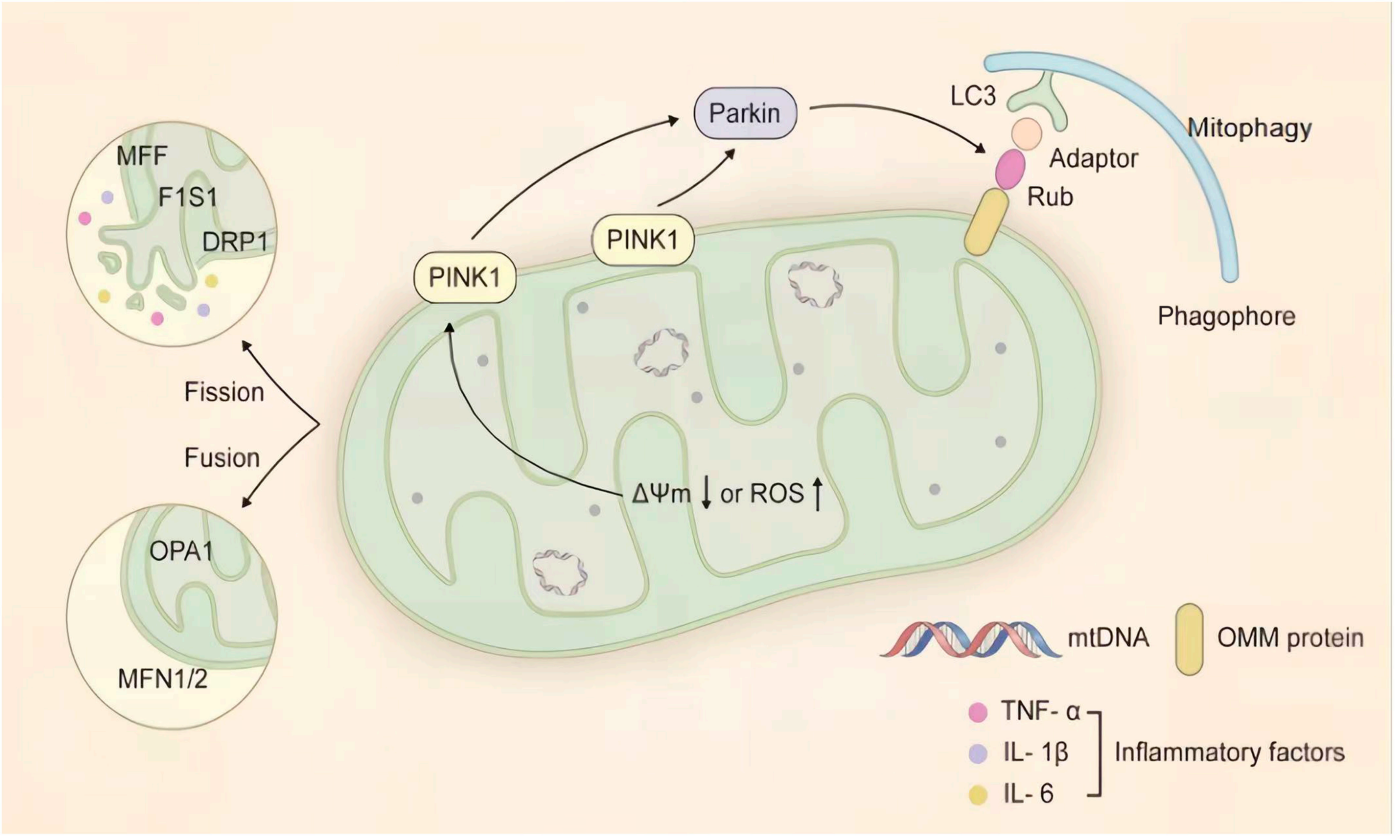
Schematic diagram of mitochondrial structure and quality control. The central body displays the double-membrane structure of mitochondria, including the outer membrane, the inner membrane folds forming cristae, and the matrix containing mtDNA. The circle to the left illustrates the dynamic balance between fusion and fission: the fusion process is regulated by MFN1/2 (outer membrane fusion) and OPA1 (inner membrane fusion), while the fission process is mediated by proteins, such as DRP1, FIS1, and MFF. A decrease in mitochondrial membrane potential (ΔΨm↓) or an increase in ROS activates the PINK1/Parkin signaling pathway: PINK1 accumulates on damaged mitochondria and recruits Parkin, which then initiates mitophagy through ubiquitination modifications, and ultimately forms phagosomes to clear damaged mitochondria. Lower right corner: the release of inflammatory factors (TNF-α, IL-1β, and IL-6) owing to mitochondrial dysfunction, suggesting their pathological role in NDDs.

Remarkably, mitochondria possess self-regulatory capabilities. The core function of mtDNA in encoding subunits of the respiratory chain lies in the precise regulation of the assembly and activity of respiratory chain complexes, and thereby ensures that mitochondria can rapidly and efficiently supply energy according to the constantly changing energy demands of neurons [21]. To maintain functionality, mitochondria rely on a dual quality-control system. One part involves the dynamic balance between fusion and fission. Fusion, mediated by MFN1/2 (outer membrane) and OPA1 (inner membrane), helps stabilize mtDNA and repair damage. Fission, regulated by DRP1, FIS1, and MFF, segregates dysfunctional segments [22]. Mitochondrial fusion and fission dynamics not only facilitate transport but also play a direct role in maintaining mitochondrial network health, mtDNA homogenization, and the isolation of damaged portions [23]. In neurons, which are highly dependent on a continuous supply of energy and metabolic homeostasis, this dynamic process is crucial for the removal of damaged mitochondria, the maintenance of energy metabolic balance, and the prevention of neurodegenerative changes [24]. Mitophagy constitutes a targeted degradation process for damaged mitochondria. When membrane potential drops, PINK1 accumulates on the outer membrane, recruiting Parkin, which ubiquitinates damaged mitochondria, targeting them for autophagic removal [25]. In NDDs, these quality-control mechanisms frequently fail, leading to the accumulation of dysfunctional mitochondria [26], impaired ATP synthesis, and release of pro-inflammatory factors such as tumor necrosis factor alpha (TNF-α), interleukin-1 beta (IL-1β), and interleukin-6 (IL-6), and thereby exacerbate neuroinflammation [27] (Fig. 2).

### Mitochondrial energetic functions

Mitochondria, the cell’s primary energy source, are critical for maintaining physiological function. The mitochondrial respiratory chain, comprising 5 complexes, drives cellular energy production [28]. Neurons have a high energy demand and primarily rely on OXPHOS to produce ATP. However, the specific ratio may vary depending on the type of neuron and its physiological state [29]. Electron transport via complexes I, III, and IV pumps protons into the intermembrane space, to create a transmembrane gradient. Complex V uses this electrochemical gradient to synthesize ATP [30]. In NDDs, ATP levels in brain tissue are significantly reduced. Impaired ATP synthesis is a major contributor to disease progression, and there is a significant decrease in the activity of mitochondrial complexes [14,31].

Beyond energy production, mitochondria regulate redox balance and calcium homeostasis. Reactive oxygen species (ROS), primarily from complexes I and III, act as signaling molecules at moderate levels; however, at excessive levels, they induce oxidative stress and damage [32,33]. Neurons are particularly vulnerable to oxidative stress owing to high polyunsaturated fatty acid (PUFA) content and limited antioxidant defenses [34]. Mitochondria can finely balance the production and clearance of ROS by regulating the electron flow of the respiratory chain, modulating membrane potential, and employing a robust internal antioxidant enzyme system (i.e., SOD2, GPx/Prx, and GSH/Trx) to maintain signaling ROS levels [35]. Furthermore, mitochondria also buffer intracellular calcium via voltage-dependent anion channel and the mitochondrial calcium uniporter (MCU). Under pathological conditions, calcium overload opens mitochondrial permeability transition pores (mPTPs), which causes membrane potential to collapse and triggers apoptosis [36].

These 3 functions—ATP production, ROS regulation, and calcium buffering—are central to mitochondrial control of cellular metabolism and homeostasis. Their dysregulation underlies the pathogenesis of NDDs [37,38].

### Mitochondrial regulation of substrate metabolism

Mitochondria serve as the central hub for cellular energy metabolism and are critical for the integration and regulation of glucose–lipid metabolism. In glucose metabolism, pyruvate derived from glycolysis is converted into acetyl-CoA by the pyruvate dehydrogenase complex (PDC) in the mitochondria. Acetyl-CoA enters the TCA cycle, producing nicotinamide adenine dinucleotide (NADH) and FADH_2_, which drive the mitochondrial electron transport chain (ETC) to generate ATP [39]. The brain accounts for approximately 20% of total glucose utilization, reflecting the high metabolic demands of the nervous system [40]. In AD, impairments in glucose metabolism have been observed, including reduced glucose utilization in the temporoparietal cortex, decreased PDC activity, and down-regulated expression of key TCA cycle enzymes—highlighting the profound influence of mitochondrial dysfunction on glucose metabolism and NDDs [41].

Furthermore, mitochondria play a critical role in lipid metabolism. They convert long-chain fatty acids into acetyl-CoA through β-oxidation [30]. Although glucose is the preferred energy substrate for the nervous system, under pathological conditions, β-oxidation of fatty acids becomes an important alternative energy source [42]. In AD, the dysfunction of MAMs is particularly pronounced [43]. MAMs serve as dynamic contact points between the endoplasmic reticulum and mitochondria, acting as key platforms for lipid biosynthesis, transport, and calcium signaling. The dysfunction of MAMs in AD leads to profound alterations in phospholipid metabolism, particularly impairing the synthesis and transport of phosphatidylserine (PS) [44]. The deficiency or abnormal distribution of PS disrupts the physicochemical properties of neuronal membranes, impairs synaptic function, and may promote neuronal vulnerability [45]. Furthermore, mitochondria regulate systemic metabolism by producing intermediates such as citrate and succinyl-CoA through the TCA cycle. Mitochondrial metabolites also possess signaling and epigenetic functions: acetyl-CoA regulates histone acetylation, while α-ketoglutarate influences DNA demethylation, thereby modulating transcription [46]. Consequently, lipid metabolic imbalances caused by mitochondrial dysfunction (including MAM abnormalities and TCA cycle disturbances) further compromise the fluidity of neuronal membranes, the structure and function of signaling microdomains (such as lipid rafts), energy homeostasis, and the activity of neuroprotective signaling pathways. This constitutes an important pathological basis for the occurrence and development of NDDs [47,48].

In NDDs, metabolic imbalance driven by mitochondrial dysfunction becomes a central pathogenic feature. This metabolic imbalance exhibits early complex bidirectional interactions between glucose and lipid metabolism [49,50]. In summary, to gain an in-depth understanding of these complex pathological processes, it is essential to explore the underlying molecular mechanisms [51]. These mechanisms involve the dynamic regulation of fusion/fission, the AMPK/mTOR/SIRT1 (Sirtuin 1) signaling pathway closely related to energy sensing, and the PINK1/Parkin pathway responsible for recognizing and clearing damaged mitochondria. These interconnected signaling networks not only lay the groundwork for understanding mitochondrial dysfunction in NDDs but also provide multiple potential therapeutic targets for developing intervention strategies. The “AMPK/MTOR pathway in mitochondrial metabolic dynamics” section elucidates specific molecular mechanisms and potential therapeutic targets.

## Cellular Metabolic Reprogramming and Inflammatory Circuitry in NDDs

### Neuronal metabolic dysfunction

NDDs are characterized by central nervous system insulin resistance—a key pathological feature and a commonality across various conditions that is frequently referred to as “type 3 diabetes” [52] . Both type 1 and type 2 diabetes importantly impact the nervous system. In type 1 diabetes, prolonged hyperglycemia can lead to neuropathy, evinced as peripheral neuropathy and autonomic dysfunction, primarily owing to metabolic imbalance and vascular damage resulting from insulin deficiency [53]. In contrast, in type 2 diabetes, the presence of insulin resistance increases the occurrence of inflammatory responses in the spinal cord and brain, promoting oxidative stress and functional impairment of neurons [54]. Furthermore, type 2 diabetes is also associated with cognitive decline and an increased risk of AD [55]. The brain’s insulin signaling pathway, from a molecular standpoint, is critical for neuronal survival, synaptic plasticity, and cognitive function [56]. Under normal physiological conditions, insulin binding to the insulin receptor (IR) activates the IR substrate (IRS), which culminates in the activation of the PI3K–Akt pathway [57]. The translocation of GLUT4 to the cell membrane promotes increased glucose uptake. In NDDs, this finely tuned regulatory network undergoes important alteration. In the brain tissue of patients with AD, diminished or aberrantly phosphorylated insulin signaling components (IR, IRS-1, and PI3K) are observed, which results in impaired insulin signaling [58]. The binding of insulin to its receptor is hindered by competition with amyloid-beta (Aβ), thereby blocking downstream signaling. These changes collectively reduce the expression and membrane translocation of GLUT4 and GLUT3 in neurons, and leads to impaired glucose uptake [59]. Other NDDs, such as PD and HD, also exhibit distinct mechanisms of insulin resistance; however, their underlying mechanisms may differ from those of AD. For instance, insulin resistance in PD may be associated with the loss of dopaminergic neurons and inflammatory responses [60], whereas in HD, it may be linked to the abnormal aggregation of the huntingtin protein [61]. Upon its onset, insulin resistance leads to a comprehensive restructuring of glucose metabolism. It is associated with decreased expression and activity of key glycolytic enzymes, such as hexokinase, phosphofructokinase, and pyruvate kinase, thus reducing the efficiency of glucose utilization [62]. Reduced pyruvate dehydrogenase activity in the brain tissue of patients with NDD further impairs pyruvate entry into the TCA cycle. Thus, decreased energy production may drive the conversion of pyruvate to lactate, which contributes to local acidosis and creates a metabolically hostile environment for neurons [63].

The persistent high-glucose environment caused by insulin resistance promotes the formation of advanced glycation end products (AGEs) [64], which represent a critical molecular link between metabolic dysfunction and neuronal damage. AGEs are neurotoxins that bind to the receptor for AGEs (RAGE) and activate inflammatory signaling pathways, including NF-κB, which increase the levels of pro-inflammatory cytokines such as IL-1β, TNF-α, and ROS [65]. Recent studies have further elucidated the multifaceted pathogenic roles of AGEs in NDDs. Their activation of the p38 MAPK and JNK pathways enhances Bax expression but reduces Bcl-2 levels, thereby facilitating neuronal apoptosis. Moreover, AGEs interact with cytoskeletal proteins, such as Tau, to alter their structure and function and accelerate the formation of the neurofibrillary tangle (NFT) [66]. AGEs exacerbate neuronal energy metabolism deficits by impairing mitochondrial function, reducing mitochondrial membrane potential, decreasing ATP synthesis, and increasing mitochondrial calcium overload and oxidative stress [67].

Insulin resistance, beside disrupting glucose metabolism, also reprograms lipid metabolism, and this is characterized by increased fatty acid synthesis and reduced β-oxidation efficiency [68]. In NDDs, this metabolic reprogramming disrupts the balanced utilization of energy substrates in neurons and thereby exacerbates energy deficits [69]. Increased ceramide synthesis is a hallmark of insulin resistance. Ceramide is neurotoxic and further impairs insulin signaling, establishing a self-perpetuating detrimental cycle [70]. Simultaneously, changes in membrane phospholipid composition influence ion channel activity and synaptic transmission, while dysregulated cholesterol metabolism is closely linked to Aβ production and clearance [71]. Together, these lipid metabolic anomalies and glucose dysregulation synergistically drive neurodegenerative pathology [72,73]. These neuronal metabolic dysfunctions set the stage for broader cellular responses, particularly from the brain’s resident immune cells.

### Glial metabolic reprogramming

Under conditions of neuronal metabolic stress, microglia and astrocytes experience important alterations in their basic metabolic profiles, which fundamentally shifts their functional states and contributes to disease progression [74]. Microglia, the brain’s resident immune cells, are particularly sensitive to metabolic fluctuations and respond by undergoing metabolic–phenotypic coupling [75]. Reduced glucose utilization efficiency and exposure to neuronal stress signals force microglia to shift from an anti-inflammatory M2 phenotype reliant on OXPHOS to a pro-inflammatory M1 phenotype dependent on glycolysis [76]. This metabolic transition is not merely a consequence but an active driver of neuroinflammation. The shift to glycolytic metabolism enables rapid ATP production necessary for immune activation while simultaneously increasing lactate production and creating a more acidic microenvironment. This metabolic–immune coupling substantially increases the production of pro-inflammatory cytokines, including TNF-α, IL-1β, and IL-6, which further propagate neuronal damage [77].

Astrocytes, as key metabolic support cells for neurons, undergo equally metabolic reprogramming that compromises their neuroprotective functions [78]. Under normal conditions, astrocytes provide crucial metabolic support through the astrocyte-neuron lactate shuttle, where they metabolize glucose to lactate and transfer it to neurons for energy production [79]. However, under conditions of insulin resistance and metabolic stress, astrocytic glucose metabolism becomes impaired. This metabolic dysfunction not only diminishes their capacity to support neuronal energy demands but also transforms them from metabolic allies into potential contributors to brain pathology [80]. The brain’s inflammatory response is further intensified by the high-glucose environment, which is characteristic of metabolic reprogramming, primarily through 2 mechanisms: (a) Hyperglycemia directly promotes NLRP3 inflammasome assembly and activation, thereby increasing the maturation and secretion of IL-1β and IL-18 [81]. The NLRP3 gene encodes an important intracellular multiprotein complex known as the inflammasome, which plays a crucial role in the host’s immune response to pathogens and damage stimuli. NLRP3 activation induces the production of downstream cytokines, which promotes the occurrence of inflammatory responses [82]. (b) AGEs bind to RAGE and activate the NF-κB signaling pathway and enhance the transcription of various pro-inflammatory genes [83]. Mitochondrial dysfunction leads to a reduction in α-ketoglutarate (α-KG) and a decrease in the activity of glutamine synthetase in astrocytes, which together disrupt the glutamate–glutamine cycle, triggering excitotoxicity. Moreover, this weakens α-KG-dependent epigenetic regulation and glutathione synthesis, and exacerbates oxidative damage and worsens the energy crisis in neurons [84].

Importantly, glial metabolic reprogramming extends beyond glucose metabolism to encompass lipid handling. Notable alterations occur in key enzyme systems responsible for long-chain fatty acid oxidation, including diminished expression and activity of CPT1, LCAD, and HAD in both microglia and astrocytes [85]. This reduction in lipid oxidative capacity creates a dual burden: direct impairment of cellular ATP synthesis and the abnormal accumulation of lipid metabolic intermediates, most notably long-chain acylcarnitines and ceramides [86]. These lipid intermediates further disrupt cellular function and contribute to the inflammatory milieu that characterizes neurodegeneration [87]. The metabolic reprogramming of glial cells thus creates a bridge between initial neuronal dysfunction and the broader inflammatory cascade that defines disease progression.

### Inflammation-mediated metabolic feedback

The metabolic dysfunction in neurons and the subsequent reprogramming of glial cells culminate in the establishment of a self-perpetuating inflammatory–metabolic cycle that drives disease progression. This phase represents the transition from localized cellular dysfunction to a brain-wide pathological network, which is characterized by a chronic low-grade inflammation known as metaflammation [88]. Metaflammation in the nervous system emerges as a consequence of the metabolic stress signals that emanate from dysfunctional neurons and activated glial cells. Unlike acute inflammatory responses to pathogens, metaflammation is characterized by chronicity, persistence, and low-grade activity, closely intertwined with ongoing metabolic dysfunction [89]. This inflammatory state is sustained by damage-associated molecular patterns (DAMPs) that are released from metabolically compromised cells, including mtDNA, ATP, and oxidized lipids, which are recognized by toll-like receptors and nucleotide-binding oligomerization domain-like receptors on glial cells. The recognition of these metabolic danger signals activates the NF-κB and MAPK signaling pathways, amplifies the inflammatory response, and creates a positive feedback loop [90].

Central to this pathological cycle is the reciprocal relationship between mitochondrial dysfunction and inflammation. Metabolically stressed mitochondria become both sources and targets of inflammatory damage. Impaired mitochondria then release DAMPs and generate excessive ROS, which activate inflammatory pathways [91]. In contrast, inflammatory cytokines such as TNF-α and IL-1β directly impair mitochondrial respiratory chain complexes, disrupt mitochondrial membrane potential, and interfere with mitophagy—the cellular process that is responsible for removing damaged mitochondria [92]. This bidirectional relationship creates a particularly detrimental feedback loop where “mitochondrial damage → inflammation → further mitochondrial damage” perpetuates disease progression.

Within this inflammatory framework, lipid peroxidation emerges as a critical amplification mechanism [93]. Lipid peroxidation functions as a critical amplifier within this pathological framework. The nervous system’s high concentration of PUFAs, such as arachidonic acid and docosahexaenoic acid, makes neuronal lipid membranes especially susceptible to peroxidation by ROS and leads to the production of toxic metabolites, such as 4-hydroxynonenal (4-HNE), malondialdehyde, and isoprostanes [94]. These disturbances initiate a 3-way, self-amplifying cycle that involves lipid peroxidation, oxidative stress, and inflammation. Aldehyde compounds like 4-HNE form stable covalent adducts with proteins to induce conformational and functional changes that activate inflammatory signaling pathways—most notably NF-κB and MAPK—which, in turn, promote the transcription and release of pro-inflammatory cytokines [95]. Additionally, activated microglia and astrocytes release TNF-α and IL-1β, which enhance ROS production and inhibit endogenous antioxidant systems, thereby intensifying lipid peroxidation [96]. The ROS driving this process are primarily generated during mitochondrial dysfunction, which damages mitochondrial membranes and compromises mtDNA integrity, thereby impairing respiratory chain complex activity. The pathological cycle of “mitochondrial damage → lipid peroxidation → inflammation amplification” is a particularly detrimental feedback loop [97,98].

The inflammatory–metabolic feedback is further complicated by specific lipid imbalances that develop over time. This vicious cycle is further aggravated by imbalances in specific lipid species. In AD, for example, distinct abnormalities in cholesterol metabolism are observed in brain tissue, including elevated total cholesterol levels and a marked reduction in 24S-hydroxycholesterol, the main metabolite responsible for cholesterol clearance, indicating impaired cholesterol homeostasis [99]. These imbalances have pathological consequences, such as stimulating β-secretase activity, which promotes Aβ synthesis, while simultaneously inhibiting the protective cleavage function of α-secretase [100]. Furthermore, cholesterol accumulation alters neuronal and synaptic membrane fluidity, which affect ion-channel function and neurotransmission. The multifaceted impact of lipid imbalance on mitochondrial function is evident through complex coupling mechanisms. Excess cholesterol disrupts mitochondrial membrane fluidity and permeability, damaging the membrane bilayer and impairing ETC complexes [101]. Moreover, ganglioside GM1, a key membrane lipid, plays a role in regulating the MCU; its metabolic dysregulation directly impairs mitochondrial calcium homeostasis and energy metabolism [102]. In the process of mitophagy, phosphatidylethanolamine is a vital substrate for LC3 lipidation, and its dysregulation compromises mitochondrial quality control, facilitating the accumulation of dysfunctional mitochondria [103].

**Fig. 3.**
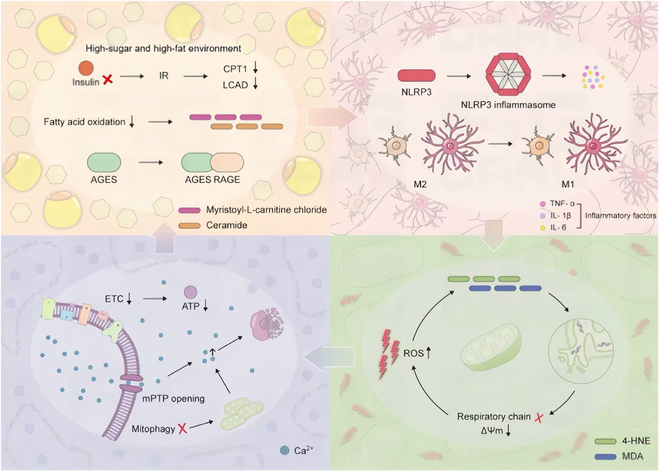
The interactive mechanisms of glucose–lipid metabolism disorders and neuroinflammation in NDDs. Top left (initiation of metabolic dysregulation): A high-sugar, high-fat environment leads to insulin resistance (IR), which results in the decreased activity of key metabolic enzymes CPT1 and LCAD. The impairment of fatty acid oxidation causes the accumulation of lipid intermediates (myristoylcarnitine and ceramide) whereas AGEs bind to RAGE, to activate downstream pathways. Top right (amplification of neuroinflammation): Activation of the NLRP3 inflammasome shifts microglia from the anti-inflammatory M2 phenotype to the pro-inflammatory M1 phenotype, leading to the release of large amounts of inflammatory factors such as TNF-α, IL-1β, and IL-6. Bottom right (oxidative stress and lipid peroxidation): Excessive ROS generation results in decreased mitochondrial respiratory chain function (ETC↓) and reduced membrane potential (ΔΨm↓), producing lipid peroxidation products 4-HNE and MDA, creating a positive feedback loop of oxidative damage. Bottom left (mitochondrial dysfunction): Opening of the mitochondrial permeability transition pore (mPTP) leads to Ca^2+^ overload, reduced ATP production, and impaired mitophagy, ultimately exacerbating the entire pathological cycle. This figure highlights the mutually reinforcing relationships among metabolic dysregulation, inflammation, oxidative stress, and mitochondrial damage.

The ultimate consequence of this inflammatory–metabolic feedback is a comprehensive disruption of brain homeostasis that manifests in distinct temporal phases. In the early phase, insulin resistance and fatty acid oxidation defects create neuronal energy deficits that trigger cellular stress responses and initial glial activation [104]. The intermediate phase is characterized by inflammation amplification, where sustained metabolic disturbances drive microglial M1 polarization and NLRP3 inflammasome activation, resulting in cytokine storm-like conditions within the brain parenchyma [105]. The late phase involves irreversible structural damage, where prolonged exposure to inflammatory mediators and lipid peroxidation products results in synaptic loss, neuronal death, and the accumulation of pathological protein aggregates [106]. This progressive pathological mechanism reveals how initial metabolic dysfunction in individual neurons can escalate into brain-wide inflammatory pathology through glial cell involvement and self-amplifying feedback loops. Understanding this cascade provides crucial insights for developing therapeutic strategies that target multiple points in the metabolism–inflammation axis to potentially interrupt the cycle before irreversible damage occurs (Fig. 3).

## NDDs and Mitochondrial Dysfunction

In NDDs, mitochondrial dysfunction and disturbances in energy metabolism are key pathological features. These mechanisms exhibit differences in AD, PD, and HD. In AD, the metabolic disturbances of mitochondria are primarily associated with the deposition of Aβ protein and the abnormal phosphorylation of tau protein. Aβ can bind to mitochondrial membrane proteins, leading to a decrease in mitochondrial membrane potential, which, in turn, triggers energy production deficits and an increase in ROS. This functional decline makes the neurons of AD patients more susceptible to oxidative stress, exacerbating cell death and cognitive decline [107]. In PD, the metabolic disturbances of mitochondria are closely related to the loss of dopaminergic neurons. In the mitochondria of patients with PD, α-synuclein aggregates to form Lewy bodies, which directly impact mitochondrial biogenesis and energy metabolism. The impairment of mitochondrial function results in insufficient energy supply to meet neuronal demands, which further decreases intracellular ATP levels, synaptic dysfunction, and reduced motor abilities [108]. In HD, mitochondrial dysfunction primarily originates from the direct interaction of the mutated huntingtin protein with mitochondria, inducing damage to the mitochondrial membrane and impairing energy production [109]. Furthermore, patients with HD frequently exhibit reduced mitophagy capacity that prevents the timely clearance of damaged mitochondria and further exacerbates metabolic disturbances within the cell [110].

In summary, although AD, PD, and HD all exhibit mitochondrial dysfunction, there are important differences in the specific metabolic disorder mechanisms. Understanding these differential mechanisms is crucial for developing personalized treatment strategies customized to specific diseases (Table 1).

**Table 1.** Comparative analysis of mitochondrial dysfunction mechanisms and therapeutic targets in NDDs

Diseases	Pathological protein	Respiratory chain complex impairment	Specific molecular mechanisms	Metabolic features	Therapeutic targets	References
AD	Aβ aggregates, hyperphosphorylated tau protein	Complex IV	• Aβ binding to alcohol dehydrogenase (ABAD) • Respiratory chain Complex IV suppression • mPTP sensitization • Disruption of nuclear-encoded protein import	• Hypometabolism in temporoparietal cortex • Pyruvate dehydrogenase complex (PDC) impairment • Down-regulation of TCA cycle enzymes	• β/γ-Secretase inhibitors • Mitochantioxidants • PINK1/Parkin pathway activators	[111,112,114,116,117]
PD	α-Synuclein aggregates	Complex I	• α-Synuclein-cardiolipin interaction • Acceleration of pathological aggregation • Proteasomal and autophagic inhibition • Ca^2+^ dyshomeostasis	• Substantia nigra-specific ATP depletion • Elevated ROS production • Mitochondrial membrane potential (ΔΨm) collapse	• PINK1 kinase enhancers • Cardiolipin modulators • Complex I protectants	[119–121,123,128]
HD	Mutant huntingtin protein (mHTT)	Complex I–IV	•mHTT-mitochondrial outer membrane association • Transcriptional repression of PGC-1α • Fission promotion and fusion impairment • Cytochrome c (Cyt c) efflux	• Generalized glucose hypometabolism in striatal and cortical regions • Systemic metabolic collapse	• PGC-1α activators •Mitochondrial dynamics modulators •Anti-apoptotic agents	[130,133–135]

### Alzheimer’s disease

The prevalent neurodegenerative disorder, AD, is typified by a gradual reduction in cognitive function and memory, along with the presence of Aβ plaques and NFTs within the brain. Problems in mitochondrial and energy metabolism are observed early in the progression of AD and precede the appearance of classical pathological markers. These findings introduce a new perspective for interpreting the progression of AD [111].

A comprehensive multidimensional analysis of the pathological signs demonstrated varied and intricate mitochondrial damage in patients with AD. Reduced in size, mitochondria display broken cristae structure and compromised membrane integrity. Its function is associated with decreased mitochondrial membrane potential, decreased ATP synthesis, increased ROS levels, and calcium imbalance. At the molecular level, the brain tissue of patients with AD demonstrates a substantial reduction in the activity of mitochondrial respiratory chain complexes, especially complex IV, along with an increase in mtDNA mutations, an imbalance in mitochondrial dynamics (manifested by reduced fusion and increased fission), and compromised mitophagy [112]. Remarkably, pronounced mitochondrial alterations are predominantly observed in cognitive regions, such as the hippocampus and cortex, and are closely associated with memory issues. The regional damage pattern offers a coherent explanation for the primary early symptom of AD, namely, memory impairment [113]. A self-perpetuating cycle of mitochondrial dysfunction and AD-specific pathology forms the core pathological foundation of AD progression. Aβ impairs mitochondrial function through several mechanisms: its localization to mitochondria, binding to ABAD, and inhibition of complex IV, all of which increase ROS levels. Additionally, Aβ promotes the opening of the mPTP, resulting in calcium overload and cytochrome c release. Overexpression of amyloid precursor protein leads to its accumulation on the mitochondrial membrane, where it blocks the import of nuclear-encoded mitochondrial proteins, further disrupting mitochondrial function [114]. Furthermore, the promoting effect of Aβ is not limited to direct damage to mitochondrial function but also involves alterations in mitochondrial morphology. The overexpression of Aβ induces adverse mitochondrial fusion and fission, which nearly disrupts the mitochondrial network. Furthermore, Aβ can activate intracellular calcium channels, which leads to calcium overload that further damages mitochondrial energy metabolism and induces apoptosis. This increased calcium load induces ROS production within the mitochondria, which exacerbates oxidative damage and creates a vicious cycle [115].

The promotion of Aβ and tau pathology by mitochondrial dysfunction is multifaceted. Energy deficits and oxidative stress enhance the activity of β-secretase and γ-secretase, thereby increasing Aβ production. ROS generated by dysfunctional mitochondria activate kinases, such as GSK-3β, CDK5, and JNK, which contribute to the abnormal phosphorylation of tau. In addition, defective mitochondrial proteases impair autophagy and proteasomal degradation, reducing the clearance of Aβ and tau proteins [116,117].

Abnormalities in glucose metabolism represent another critical pathological hallmark of AD, further contributing to disease progression. Positron emission tomography (PET) imaging studies have demonstrated a marked reduction in the cerebral metabolic rate of glucose in patients with AD, with the extent of this decline correlating with cognitive impairment severity. Notably, reductions in brain glucose metabolism appear prior to the clinical onset of symptoms and before substantial Aβ accumulation, suggesting that alterations in energy metabolism occur in the early stages of AD [118]. This biomarker holds important value for early diagnosis and timely therapeutic intervention.

### Parkinson’s disease

PD, the second most common neurodegenerative disorder, is characterized by the progressive degeneration of dopaminergic neurons in the substantia nigra and the accumulation of Lewy bodies. The central role of mitochondrial dysfunction in PD pathogenesis has been underscored by 2 pivotal findings. First, mitochondrial toxins, such as mPTP, selectively damage dopaminergic neurons in the substantia nigra, producing PD-like symptoms. Second, several PD-associated genes—including *PINK1*, *Parkin*, *DJ-1*, and *LRRK2*—encode proteins critical for the regulation of mitochondrial function. These findings collectively support the conclusion that mitochondrial dysfunction constitutes a fundamental mechanism underlying the development and progression of PD [119,120].

The most prominent and consistent mitochondrial abnormality that is observed in PD is complex I dysfunction—a finding that has been corroborated in numerous studies. In patients with PD, mitochondrial complex I activity is reduced by approximately 30% to 40% specifically in the substantia nigra, while the activity of respiratory chain complexes II to V remains relatively stable [121]. This selective complex I deficiency leads to several pathogenic consequences, including reduced ATP production, increased ROS generation, and decreased mitochondrial membrane potential, which ultimately results in neuronal death. The selective vulnerability of dopaminergic neurons in the substantia nigra is primarily attributed to this region-specific mitochondrial dysfunction [122].

Recent research reveals that cardiolipin plays a crucial role not only in maintaining the integrity of mitochondrial membranes but also in facilitating the formation of aggregates of α-synuclein. By binding to α-synuclein, cardiolipin regulates its structure and function, promoting its pathological aggregation. This interaction mechanism has shown importance in NDDs such as PD, which indicates that cardiolipin may represent a novel mechanism in the aggregation and pathological progression of α-synuclein [123]. Furthermore, alterations in cardiolipin are closely associated with intracellular Ca^2^ dynamics, with studies indicating that reduced levels of cardiolipin may accelerate the aggregation of α-synuclein. This finding provides new insight for interventional strategies that target cardiolipin, suggesting that modulating cardiolipin metabolism could potentially slow the progression of α-synuclein-related pathology [124]. Moreover, complex I-related mitochondrial deficits also contribute to the pathological accumulation of α-synuclein, establishing a central cascade in PD progression. This process unfolds through multiple mechanisms: first, impaired complex I function reduces ATP levels and elevates ROS production, which together inhibit the proteasome and the autophagy–lysosome system, thereby diminishing α-synuclein clearance; second, oxidative stress promotes the oxidative modification of α-synuclein, increasing its aggregation propensity; finally, mitochondrial dysfunction induces calcium dysregulation, and calcium overload enhances α-synuclein binding to membranes, further accelerating its aggregation. This pathogenic cycle—"mitochondrial dysfunction → oxidative stress → protein aggregation”—constitutes the core of PD pathology, bearing similarities to that of AD, while maintaining disease-specific features [125,126].

Thus, it is crucial to emphasize the selectivity and specificity of mitochondrial dysfunction in PD. Unlike other neurodegenerative disorders, PD is characterized by a targeted deficiency in complex I, with other respiratory chain complexes largely unaffected. This region-specific damage pattern reflects the unique metabolic demands of dopaminergic neurons [127]. Recent studies indicate that the activation of PINK1 can enhance mitochondrial membrane potential to facilitate the process of mitophagy, and thereby prevent the accumulation of damaged mitochondria [128]. Moreover, mutations in *PINK1* and *Parkin*, genes linked to familial forms of PD, impair mitochondrial quality control mechanisms. Deficiencies in these proteins result in inadequate removal of damaged mitochondria, exacerbating mitochondrial dysfunction and illustrating the molecular basis of genetic susceptibility to PD. In-depth research into these mechanisms reveals the potential therapeutic target of PINK1 in NDDs [129].

### Huntington’s disease

An autosomal dominant neurodegenerative disorder, HD, is caused by a CAG trinucleotide repeat expansion within the *HTT* gene. The condition is characterized by the selective degeneration and loss of medium spiny neurons within the striatum, accompanied by choreiform involuntary movements, cognitive impairments, and psychiatric manifestations. HD’s genetic profile constitutes a unique model for investigating the impact of mitochondrial dysfunction on NDDs [130]. The nature of mitochondrial dysfunction in HD is notable for its extensive severity, contrasting with the more localized damage observed in AD and PD. Patients with HD and animal models exhibit substantial structural and functional mitochondrial anomalies, as documented pathologically [131]. Swelling is observed in the mitochondria of striatal neurons in patients with HD, along with disrupted cristae and compromised membrane integrity. Functional impairments include a decrease in mitochondrial membrane potential, reduced ATP synthesis, diminished calcium buffering capacity, and increased production of ROS [132]. Patients with HD demonstrate reduced complex I activity, as well as marked reductions in complexes II, III, and IV, suggesting a broader impairment of the mitochondrial respiratory chain compared to that observed in PD. This widespread mitochondrial dysfunction highlights the complexity and severity of HD’s pathological processes [133].

The mHTT protein impairs mitochondrial function through multiple pathways, establishing the molecular foundation for HD progression. The primary disease mechanism involves mHTT binding to the outer mitochondrial membrane, increasing its permeability and promoting the release of cytochrome c, which leads to neuronal apoptosis [134]. Additionally, mHTT suppresses the expression and activity of PGC-1α, a transcriptional co-activator essential for mitochondrial biogenesis and energy metabolism, thereby directly impairing mitochondrial regeneration and repair. Furthermore, mHTT affects mitochondrial dynamics by enhancing fission and inhibiting fusion, resulting in fragmentation of the mitochondrial network [135]. These combined molecular mechanisms exacerbate energy metabolism dysfunction in HD, leading to a systemic metabolic collapse from the molecular to the cellular level [136].

**Fig. 4.**
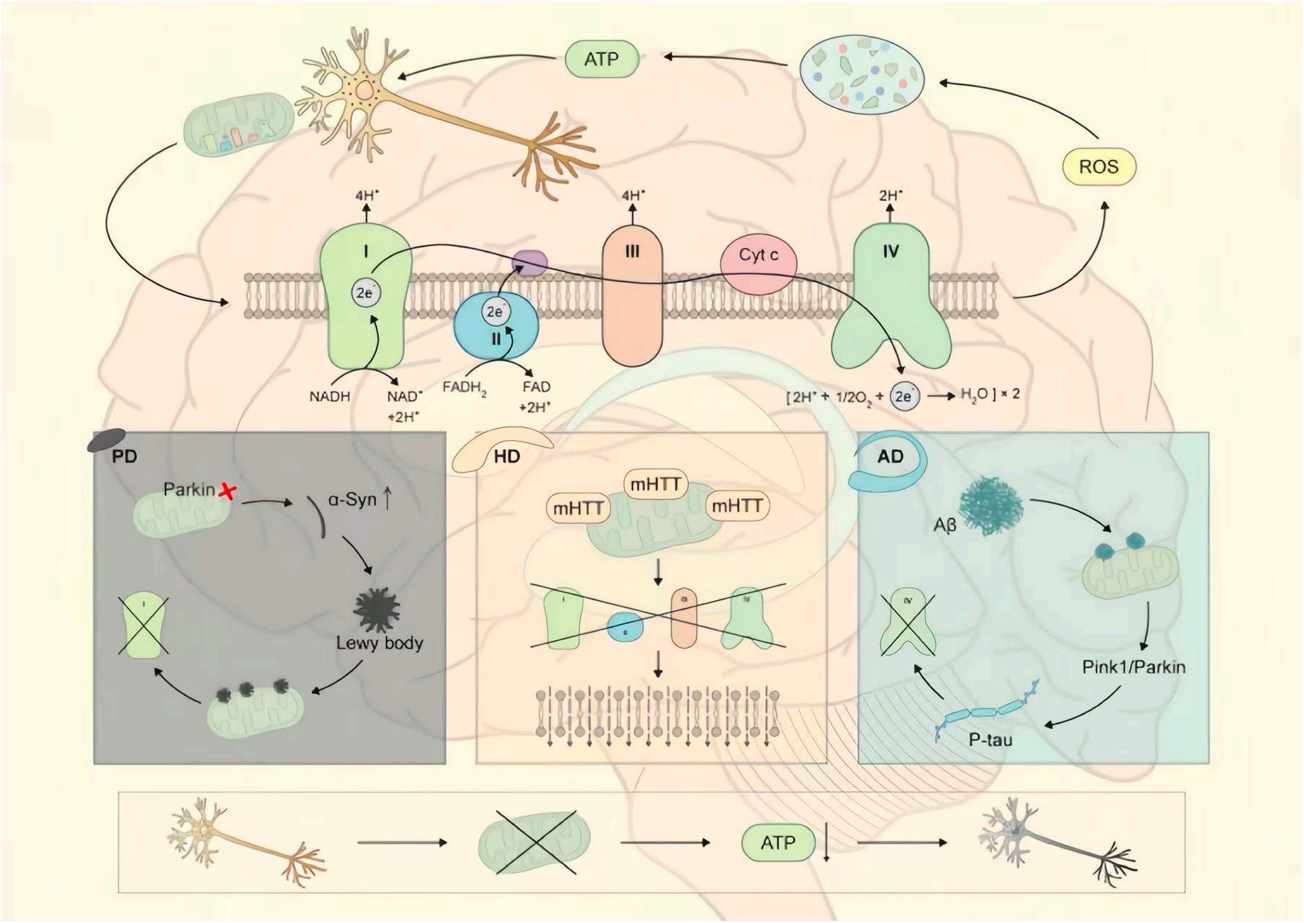
Differences in mitochondrial dysfunction among three NDDs. The upper part shows the electron transport process of normal mitochondrial respiratory chain complexes I to IV, which generates ATP through the oxidation of NADH and FADH_2_ while producing a small amount of ROS. The 3 modules at the bottom display the specific pathological mechanisms of each disease: PD module (left): Dysfunction of Parkin leads to the aggregation of α-synuclein forming Lewy bodies, primarily damaging complex I and causing mitochondrial dysfunction; HD module (middle): Mutant huntingtin (mHTT) directly impairs mitochondrial membrane integrity, and leads to the overall dysfunction of the entire respiratory chain complex; AD module (right): The aggregation of Aβ and hyperphosphorylation of tau protein (P-tau) act synergistically, to primarily affect complex IV while interfering with PINK1/Parkin-mediated mitochondrial quality control. The flowchart at the bottom summarizes the common consequences of mitochondrial dysfunction: Normal neurons → Damaged mitochondria → Reduced ATP production → Neuronal degeneration. This figure highlights the differences in pathological proteins, damaged complexes, and molecular mechanisms among the 3 diseases.

Distinctive features and clinical relevance are observed in HD’s energy metabolism due to its pathological basis. Evidence from PET scans and related imaging modalities reveals a significant reduction in glucose metabolism in patients with HD, particularly in the striatum and various cortical regions, reflecting the widespread nature of HD pathology. Notably, these metabolic abnormalities appear before clinical symptom onset and are detectable in asymptomatic gene carriers. Thus, disruptions in energy metabolism represent an early phase in HD progression, serving as a critical biomarker for early detection and therapeutic intervention [137]. Compared to AD and PD, HD displays earlier onset, a broader range, and more severe energy metabolism deficits, due to mHTT’s multiple direct deleterious effects on mitochondria [138] (Fig. 4)

## Mitochondrial Function and Glucose–Lipid Metabolism

The principal metabolic control hub, the mitochondria, sustains neuronal energy and glucose–lipid metabolic balance via complex signaling networks. In NDDs, dysfunction of key signaling pathways is associated with a direct reduction in mitochondrial energy production and subsequent impairment of the integration of the cellular metabolic network. The text delineates the pivotal functions of the AMPK/mTOR, PGC-1α, and SIRT1 signaling pathways in modulating mitochondrial function and glucose–lipid metabolism, and their dysregulatory patterns and interregulatory dynamics in NDDs (Table 2).

**Table 2.** Dysregulation of key signaling pathways in NDDs

Signaling pathway	Physiological function	Pathological alterations	Downstream effects	Therapeutic strategies	References
AMPK/mTOR	Energy sensing and metabolic homeostasis	• AMPK hypoactivation • mTOR hyperactivation • Homeostatic disruption	• Pathogenic protein aggregation • Mitophagy • Membrane lipid dyshomeostasis	• Metformin (AMPK activator) • Rapamycin (mTORC1 inhibitor)	[139,141,142,146,176]
PGC-1α	Master regulators of mitochondrial biogenesis	• Significant down-regulation • Transcriptional impairment • Epigenetic dysregulation	• Mitochondrial depletion • Bioenergetic inefficiency • Antioxidant defense impairment	• NAD^+^ precursor supplementation • SIRT1 activators • PGC-1α upstream modulators	[148,151,152,154,178]
SIRT1	NAD^+^-dependent deacetylases	• NAD^+^ depletion • Enzymatic activity reduction • Metabolic regulatory failure	• Insulin resistance • Lipid homeostasis disruption • Cholesterol dysmetabolism	•NMN/NR therapy • Resveratrol analogs • Next-generation activators (e.g., SRT2104)	[155,158,159,161,164]

### AMPK/MTOR pathway in mitochondrial metabolic dynamics

AMPK functions as the primary cellular energy sensor and is highly sensitive to subtle changes in the intracellular AMP/ATP ratio. Energy stress activates AMPK in cells, which subsequently alters metabolic pathways through various molecular mechanisms. AMPK promotes the activation of PGC-1α by phosphorylating it at Thr177 and Ser538, leading to increased expression of NRF1/2 and Tfam, thereby enhancing mtDNA replication and protein synthesis [139]. This regulatory mechanism allows for the rapid up-regulation of mitochondrial quantity and function to meet metabolic demands during energy scarcity [119].

AMPK plays a crucial role in metabolic reprogramming within the glucose–lipid metabolic domain. Upon activation, AMPK facilitates glucose uptake by promoting GLUT4 translocation to the plasma membrane and inhibits fatty acid synthesis through ACC1 phosphorylation [140]. Moreover, it activates CPT1, thereby enhancing fatty acid oxidation. This metabolic adaptation optimizes energy substrate utilization and reduces energy-intensive biosynthetic processes during periods of energy stress. Notably, AMPK regulation is altered in neurodegenerative disorders [141]. Studies on AD models have shown a negative correlation between AMPK activity and Aβ accumulation, suggesting that AMPK dysfunction may contribute to disease progression via disruptions in glucose and lipid metabolism [142]. Importantly, chronic stress can worsen the neuronal energy crisis by maintaining persistently high AMPK activity, which promotes excessive mitochondrial fission and autophagy, forming a detrimental cycle of “overcompensation and resultant functional failure” [143].

When AMPK is inactive, mTOR becomes the dominant regulator under energy-abundant conditions. It integrates signals from nutrient status, growth factors, and energy availability to govern cell growth, protein synthesis, and metabolic adaptation. mTORC1 activation promotes ribosome biogenesis and protein translation and also influences lipid metabolism by regulating SREBP-1c, a transcription factor central to lipid synthesis [144]. In addition, mTORC1 regulates the expression of TFAM and mitochondrial ribosomal proteins, which are critical for mitochondrial biogenesis and function [145].

A complex reciprocal inhibitory interaction exists between AMPK and mTOR. Under energy-deficient conditions, AMPK inhibits mTORC1 by phosphorylating TSC2 and Raptor, while inducing autophagy to eliminate damaged mitochondria. Conversely, in energy-rich conditions, mTOR signaling becomes active, suppressing AMPK activity and promoting anabolic metabolism and cell proliferation [146]. Neuronal metabolic homeostasis depends on maintaining this dynamic balance. In NDDs, persistent overactivation of the mTOR signaling pathway is a key pathological feature. Overactive mTOR contributes to the accumulation of abnormal proteins and impaired mitophagy, thereby exacerbating oxidative stress and energy imbalance. Additionally, aberrant mTOR activation alters neuronal membrane lipid composition and mitochondrial membrane dynamics, in part through regulation of SREBP1, affecting neurotransmission and synaptic plasticity [147].

### PGC-1α on mitochondrial energy metabolism

In the nervous system, PGC-1α, as a master transcriptional coactivator, occupies a central position in energy metabolism regulation. PGC-1α, in association with diverse transcription factors, forms complexes that modulate the transcription of mitochondrial genes, thereby facilitating the precise regulation of mitochondrial function [148].

PGC-1α, interacting with NRF1/2, peroxisome proliferator-activated receptors (PPARs), and estrogen-related receptor alpha, forms a complex transcriptional regulatory network [149]. PGC-1α’s multi-target regulatory mechanism facilitates the concurrent modulation of mtDNA replication, ETC complex assembly, TCA cycle enzyme expression, and antioxidant defense system formation. The expression level of PGC-1α in neurons directly influences the quantity, morphology, and functional status of mitochondria, thereby modulating the neuron’s energy supply and oxidative stress resistance. PGC-1α is pivotal in modulating energy substrate utilization. PGC-1α achieves glucose metabolism–fatty acid oxidation balance through its interaction with the PPAR family. Typically, PGC-1α fosters fatty acid oxidation gene expression and sustains glucose metabolism, supporting neural adaptation to energy substrates across various metabolic states [150]. The efficacy of neurons in managing fluctuations in energy demand and oxidative stress is underpinned by their metabolic flexibility. PGC-1α expression decrement is a prevalent pathological hallmark in neurodegenerative disorders. The selective ablation of PGC-1α in substantia nigra dopaminergic neurons triggers Parkinsonian mitochondrial dysfunction and neurodegeneration, emphasizing the necessity of PGC-1α for neuronal survival [151]. AD model research has confirmed a strong association between decreased PGC-1α expression and elevated mitochondrial fragmentation, reduced energy synthesis, and synaptic issues [152].

Apart from its direct regulation of mitochondrial biogenesis, PGC-1α contributes to the preservation of the mitochondrial network’s dynamic equilibrium. PGC-1α modulates the expression of the mitochondrial fusion proteins MFN1/2 and the fission protein DRP1, thereby affecting mitochondrial morphology and distribution within axons and dendrites [153]. In NDDs, PGC-1α dysfunction contributes to a disturbance in mitochondrial dynamics, which impedes the delivery of mitochondria to high-energy-consuming regions, thereby exacerbating local energy deficits. Recent studies have illuminated PGC-1α’s essential role in epigenetic regulation. PGC-1α exerts control over the synergy of mitochondrial–nuclear gene expression through histone acetylation and noncoding RNA pathways, thereby achieving a refined control of the neuronal metabolic network [154]. The finding offers a fresh theoretical basis for the PGC-1α signaling pathway as a therapeutic candidate in neurodegenerative disorders.

### SIRT1 signaling on glucose–lipid homeostasis

By deacetylating PGC-1α at sites, such as K13, K139, and K778, SIRT1 enhances its transcriptional activity, thereby establishing the SIRT1–PGC-1α regulatory axis. This axis is critical for maintaining mitochondrial function and promoting mitochondrial biogenesis [155]. Concurrently, SIRT1 facilitates the selective removal of damaged mitochondria through the deacetylation and activation of the transcription factor FOXO3a, which induces the expression of mitophagy-related genes [156]. This dual regulatory mechanism ensures the coordinated increase in mitochondrial quantity and maintenance of mitochondrial quality.

Moreover, SIRT1 modulates the function of several metabolic enzymes via direct deacetylation. Enzymes such as acetyl-CoA synthetase and pyruvate dehydrogenase, which are integral to the TCA cycle, are regulated by SIRT1, thereby influencing the efficiency of mitochondrial energy metabolism [157]. Additionally, SIRT1 enhances insulin sensitivity through deacetylation of IRS-1, improving neuronal glucose utilization—a mechanism of particular relevance in NDDs characterized by insulin resistance, such as AD [158].

Moreover, SIRT1 exerts a dual role in lipid metabolism. It suppresses fatty acid synthesis by deacetylating SREBP-1c, thereby reducing intracellular lipid accumulation. Conversely, it promotes fatty acid oxidation through activation of PPARα, increasing lipid utilization for energy [159]. Regulation of lipid metabolism is essential for preserving myelin integrity and synaptic function in the nervous system. SIRT1 also plays a key role in maintaining neuronal cholesterol homeostasis, partly through regulation of CYP46A1, a mechanism linked to Aβ production and tau phosphorylation [160]. These findings support a potential neuroprotective role for SIRT1 in the pathogenesis of AD.

NAD^+^ precursors, such as nicotinamide mononucleotide (NMN) and nicotinamide riboside (NR), have demonstrated important neuroprotective effects by promoting the synthesis of intracellular NAD^+^, enhancing mitochondrial function, and reducing oxidative stress, and thereby protecting neurons from damage. These precursors can also regulate cellular metabolism and damage repair by activating deacetylases such as sirtuins [161]. Importantly, the ability of NMN and NR to penetrate the blood–brain barrier has made them the focus of research. NMN has been shown in in vitro studies to effectively cross the blood–brain barrier via specific transport proteins (such as Slc12a8) and has demonstrated a certain level of brain bioavailability in mouse models [162]. NR, on the other hand, is believed to enter brain tissue through simple diffusion. Once inside the brain, these precursors can replenish NAD^+^ levels, to further enhance the neuroprotective effects. Recent studies have indicated that administering NMN or NR via oral or injection routes in animal models can increase brain NAD^+^ levels, which contributed to the improvement of cognitive functions associated with NDDs [163].

As a NAD^+^-dependent enzyme, SIRT1 activity is tightly regulated by intracellular NAD^+^ levels. With aging and disease progression, a gradual decline in NAD^+^ levels leads to reduced SIRT1 activity, impairing mitochondrial function and energy metabolism [159]. The cascade—"NAD^+^ reduction → decreased SIRT1 activity → mitochondrial dysfunction”—may represent a core pathogenic mechanism in aging-related NDDs. Supplementation with NAD^+^ precursors, such as NMN and NR, reportedly enhances SIRT1 activity, improves mitochondrial function and metabolic status, and exerts neuroprotective effects in various NDD models [164]. These findings provide strong justification for therapeutic strategies targeting NAD^+^ metabolism.

### Synergistic mechanism of the three in lipid homeostasis

AMPK, PGC-1α, and SIRT1 interact to maintain glucose and lipid homeostasis, forming a sophisticated integrated regulatory network. Their combined actions ensure the balance and coordination of cellular metabolism under different physiological states. Firstly, the interaction between AMPK activation and SIRT1 is crucial—AMPK is a key sensor of energy deficiency within cells. Its activation not only inhibits the mTOR signaling pathway to limit cell growth and anabolic metabolism but also enhances deacetylation by increasing SIRT1 expression. Activated SIRT1 further deacetylates PGC-1α, enhancing its activity in promoting mitochondrial biogenesis and fatty acid oxidation [165]. Secondly, the functional enhancement of PGC-1α and feedback regulation play important roles—enhanced PGC-1α not only increases mitochondrial biogenic capacity but also effectively promotes fatty acid oxidation [166]. Furthermore, PGC-1α can reduce the activity of the AMPK/mTOR pathway through a negative feedback mechanism, thereby regulating cellular metabolic processes and maintaining homeostasis under energy-sufficient conditions [167]. Lastly, SIRT1’s regulatory role on AMPK–SIRT1 enhances AMPK activity through deacetylation. This process aids in strengthening the cellular regulation of glucose and lipid metabolism during energy deficiency, which ensures that cells can timely respond to environmental changes and adjust their metabolic state [158].

**Fig. 5.**
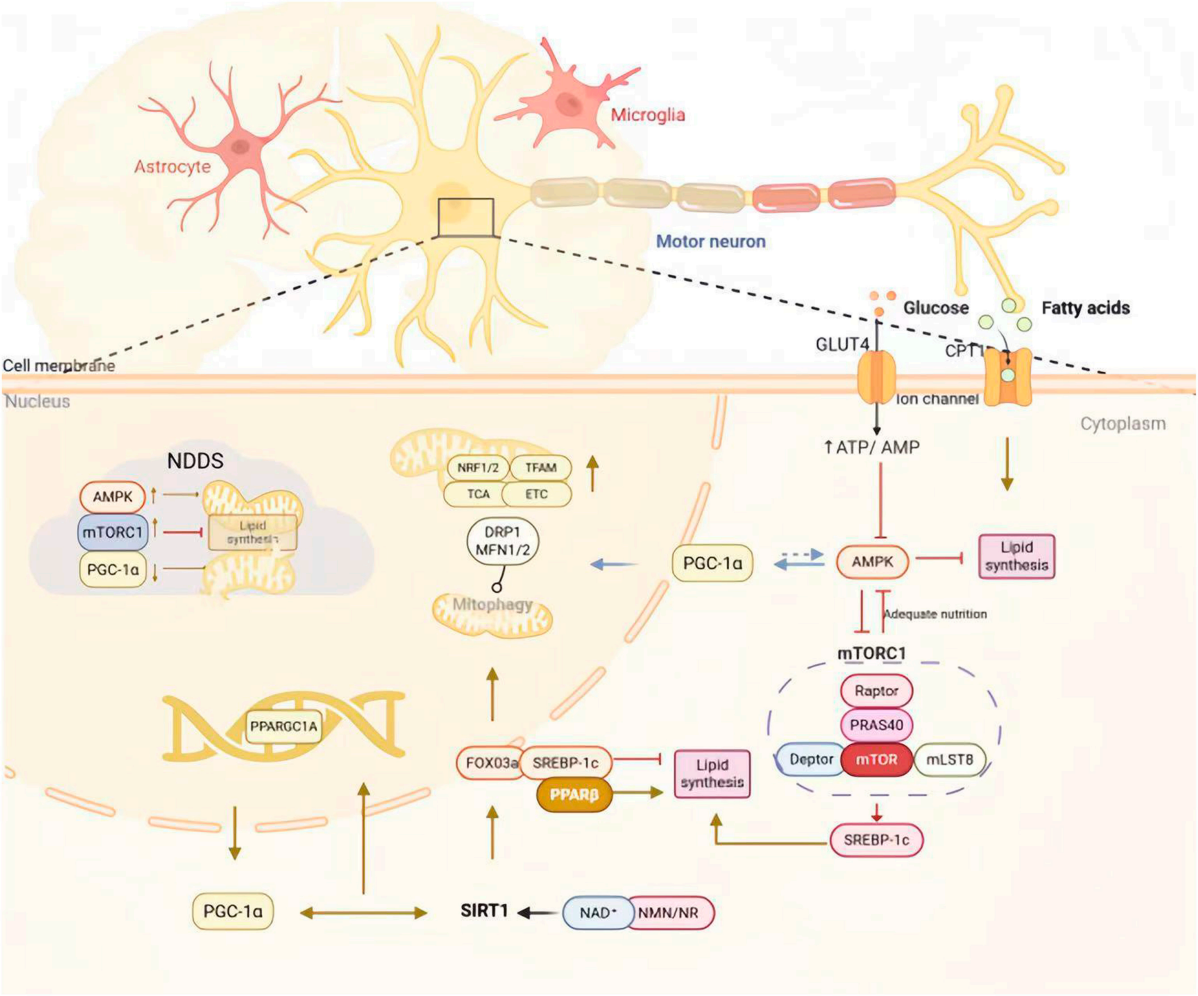
The synergistic regulation of AMPK/mTOR, PGC-1α, and SIRT1 in neuronal mitochondrial and glucose–lipid metabolism. The top panel displays neurons and their microenvironment, where GLUT4 and CPT1 on the neuronal membrane mediate the uptake of glucose and fatty acids. The intracellular signaling network is centered around the dynamic balance between AMPK and mTORC1: AMPK activation promotes GLUT4 translocation and fatty acid oxidation but inhibits lipid synthesis and suppresses mTORC1 activity; in contrast, under nutrient-rich conditions, the mTORC1 complex is activated and promotes lipid synthesis via SREBP-1c, which, in turn, feedback-inhibits AMPK. PGC-1α receives dual activation through AMPK phosphorylation and SIRT1 deacetylation, and coordinates mitochondrial biogenesis and mitochondrial dynamics. SIRT1 can be activated by NMN/NR precursors, which not only activate PGC-1α but also promote mitochondrial autophagy through FOXO3a activation, inhibit SREBP-1c, and activate transcription factors such as PPARα. The *PPARGC1A* gene in the nucleus encodes the PGC-1α protein, forming a transcriptional regulatory feedback loop. The NDDS module (to the left) indicates that the imbalance of this regulatory network is associated with disease occurrence, where excessive AMPK activation leads to abnormal mitochondrial fission, excessive mTORC1 activation inhibits autophagy and promotes abnormal protein aggregation, and the decline in PGC-1α expression reduces SIRT1 activity and collectively exacerbates mitochondrial dysfunction.

In summary, the synergistic interaction among AMPK, PGC-1α, and SIRT1 plays an important role in promoting energy metabolism. This interconnection not only effectively regulates the energy balance within cells but also plays a crucial role in responding to metabolic stress conditions, and provides new directions and targets for the potential treatment of metabolic-related diseases (Fig. 5).

## Exploring Therapeutic Strategies Targeting the Multi-Organelle MMH Network

### Regulation of mitochondrial metabolism by organelle interactions

Mitochondria do not operate in isolation; instead, they form close functional contact sites with various membranous organelles within the cell, constituting a complex interaction network. These interactions profoundly influence mitochondrial metabolic activities, thereby regulating glucose and lipid homeostasis within neurons. Firstly, the endoplasmic reticulum–mitochondria contact sites (MAMs) are dynamic structures that physically connect the endoplasmic reticulum to the outer membrane of mitochondria, to serve as critical hubs for lipid synthesis and transport, as well as Ca^2+^ signaling. The ER directly provides phospholipid precursors and Ca^2+^ to mitochondria through MAMs, which affects the lipid composition and membrane fluidity of mitochondrial membranes, which, in turn, regulates the activity of embedded metabolic enzymes and the morphology of mitochondrial cristae, driving glucose OXPHOS [16]. In AD, amyloid precursor protein (APP) and presenilin (PSEN) mutants abnormally accumulate in MAMs and thereby disrupt Ca^2+^ homeostasis and lipid transfer to promote neurotoxic Aβ generation [168]. Additionally, the accumulation of ceramide in MAMs can induce the opening of the mPTP, triggering apoptosis [169]. Secondly, lysosomes act as degradation centers that selectively remove damaged mitochondria through mitophagy, maintaining mitochondrial quality. Moreover, lysosomes are also involved in nutrient sensing and the release of metabolites (e.g., amino acids and cholesterol), among which the fatty acids derived from lipid droplets (LDs) are important substrates for mitochondrial β-oxidation [170]. Impaired lysosomal function in NDDs leads to the accumulation of LDs (increased neuronal lipofuscin) whereas limiting the availability of free fatty acids for mitochondria, and thus affecting energy supply and lipid homeostasis. Peroxisomes are responsible for initiating the β-oxidation of very long-chain fatty acids (VLCFAs), which results in the production of shorter-chain fatty acids that can be transported to the mitochondria for complete oxidation and energy production. Both processes share certain oxidative enzyme systems and cofactors [171]. Dysfunction of peroxisomes can lead to the accumulation of VLCFAs, which not only exhibits direct neurotoxicity but also competitively inhibits the β-oxidation of long-chain fatty acids in the mitochondria, reducing acetyl-CoA generation and ATP production. Additionally, the accumulation of VLCFAs impairs membrane fluidity and affects the functionality of mitochondrial membrane proteins. ROS generated by peroxisomes may also diffuse to adjacent mitochondria, exacerbating oxidative stress [172].

In summary, the collapse of neuronal glycosyl-lipid homeostasis in NDDs is not caused by a single organelle but rather results from the disruption of a multi-organelle interaction network and the imbalance of mitochondrial dynamics. Therefore, restoring the coordinated interaction between organelles and the healthy dynamics of the mitochondrial network is a key strategy for restoring glycosyl-lipid homeostasis and protecting neurons. The mitochondrial–energy metabolism–glycosyl–lipid homeostasis network is crucial for regulating “multi-organelle interactions” and “mitochondrial dynamics”. It is important to clarify that the MMH network is not isolated; it encompasses multiple organelles and various dynamic regulatory networks, influencing the interactions among multiple organelles and the processes of mitochondrial dynamics. However, the regulatory mechanisms of interactions among multi-organelles and the relationship between dynamic changes in mitochondrial morphology and function merit further investigation. This will be a key focus of future research.

### Transition from molecular insights to therapeutic concepts

NDDs essentially manifest as a gradual loss of metabolic networks, rather than merely energy deficiency or specific protein abnormalities [173]. Traditional treatment strategies primarily focus on symptom control and intervention at single pathological points, resulting in limited clinical efficacy [174]. Based on the molecular mechanism analysis of signaling pathways and multi-organelle interactions, the MMH network theory provides a systematic framework for identifying therapeutic targets in NDDs. Treatment strategies should shift from “target-specific interventions” to “reconstruction of metabolic network functionality”, restoring the overall coordination and adaptability of cellular metabolic networks by modulating multiple interconnected molecular pathways and organelles. Among these, restoring metabolic sensing capacity is more important than merely supplementing energy substrates, and maintaining network stability takes higher priority than correcting local defects [175]. However, the translation from basic research to clinical application still faces numerous challenges, necessitating the establishment of rigorous translational medicine strategies. Current clinical evidence indicates that AMPK activators, such as metformin, exhibit good safety profiles; however, their specific efficacy in NDDs still requires validation through large-scale clinical trials [176,177]. In contrast, upstream regulators of PGC-1α are more targeted, yet data on long-term safety and efficacy remain limited, necessitating cautious evaluation [178]. Furthermore, the treatment of NDDs faces challenges related to blood–brain barrier permeability, and the bioavailability and targeting efficiency of certain compounds such as NMN and NR still need optimization. Multi-target intervention strategies aimed at reshaping metabolic network functions present unprecedented challenges but also offer new possibilities for disease-modifying therapies.

Furthermore, the metabolic biomarkers associated with MMH have not yet been standardized, which limits the accuracy of efficacy assessments in clinical trials. There is still a need to establish standardized mitochondrial function testing methods, metabolic flexibility assessment indicators, and dynamic monitoring parameters to reflect changes in cellular metabolic status in real time and predict disease progression, such as variations in mitochondrial respiratory reserve capacity and the dynamic changes in the ATP/ADP ratio [161]. The development of real-time functional monitoring technologies has provided new possibilities for the dynamic assessment of treatment effects. Such technologies refer to detection methods that can continuously and noninvasively monitor metabolic functional states at the organism or cellular level, including real-time imaging of mitochondrial function based on fluorescent probes and metabolic flux analysis. The hippocampal metabolic analyzer combined with a fluorescent reporting system can dynamically monitor metabolic flux, facilitating timely adjustments and optimizations of treatment protocols [179]. However, the clinical translation of these technologies still faces practical issues such as cost control, simplification of operations, and data standardization [180]. Integrating the multi-omics analysis processes of metabolomics, proteomics, and transcriptomics can achieve a comprehensive assessment of the MMH network, but the complexity of the technology and high costs limit its widespread application [181,182] (Table 3).

**Table 3.** MMH network-related biomarkers and detection methods

Category	Specific indicators	Detection methods	Clinical significance	Standardization status	References
Mitochondrial function	• ATP/ADP ratio • Respiratory reserve capacity • Membrane potential dynamics	• Hippocampal metabolic profiling • Fluorescent probe imaging • Flow cytometry	Early disease diagnosis and therapeutic efficacy monitoring	Requiring standardization	[17,30,36,179,180]
Metabolomics	• Ceramide species (C18:0/C24:0) • Long-chain acylcarnitines • TCA cycle Intermediates	• LC-MS/MS • GC-MS • NMR	Metabolic subtyping and progression risk stratification	Partially standardized	[46,70,86,181,182]
Inflammatory markers	• TNF-α, IL-1β, and IL-6 • DAMPs (mtDNA and ATP) • Lipid peroxidation by-products	• ELISA • Multiplex immunoassay • Oxidative stress assessment	Inflammatory activity quantification and treatment response evaluation	Relatively mature	[77,90,95–97]

### Stratified therapeutic approach

Treatment strategies based on the MMH network theory must take into account the metabolic phenotypic differences among patients when developing individualized diagnostic tools and treatment plans. For early-stage patients with relatively preserved metabolic reserves, NAD^+^ precursors (e.g., NMN and NR) and AMPK activators (e.g., metformin) may have neuroprotective effects, but the optimal dosage, administration route, and treatment window still need to be confirmed through large-scale randomized controlled trials [183,184]. The primary goal at this stage is to maintain the existing metabolic flexibility, which is the ability of cells to switch between different metabolic pathways in response to environmental changes and energy demands, and thereby delay further decline in network function. Minocycline not only inhibits the activation of M1 microglia but also preserves the beneficial functions of M2 cells, while demonstrating good blood–brain barrier permeability and established safety [185]. Modified curcumin formulations target the NF-κB signaling pathway through a liposomal delivery system that effectively enhances drug bioavailability [186]. For mid-term patients with impaired metabolic function, more targeted and diverse therapeutic strategies are required, such as mTOR modulators like rapamycin analogs and mitochondrial-targeted antioxidants like SS-31 [187]. These agents demonstrate theoretical potential in improving mitochondrial function and providing neuroprotection; however, their safety and long-term efficacy need further evaluation. Diversification of metabolic substrates through supplementation of ketone bodies such as beta-hydroxybutyrate and medium-chain triglyceride oil provides alternative energy sources, reducing neuronal dependence on glucose during metabolic stress [188]. This approach may hold particular importance in phenotypes with metabolic transition disorders. When the disease progresses to late-stage multi-organ failure, the primary treatment goal is to maintain existing functions and improve quality of life. Supportive care and palliative treatment may be more realistic than aggressive disease treatment. Antioxidants and anti-inflammatory drugs targeting mitochondria may provide some symptomatic relief; however, their impact on disease progression is limited [189].

### Conclusion and future perspectives

The complexity of NDDs dictates that no single theoretical framework can fully explain all pathological phenomena. However, we believe that the loss of metabolic network flexibility is central to NDDs and that the dysregulation of glucose and lipid metabolism is not caused by a single organelle or a single target pathway, but rather by the disruption of a multi-organelle interaction network. These interactions profoundly influence mitochondrial metabolic activities and thereby regulate glucose and lipid homeostasis within neurons. Therefore, we propose a mitochondria-centered multi-organelle–energy metabolic–glucose and lipid disorder network, which is crucial for regulating “multi-organelle interactions” and “mitochondrial dynamics”. However, it is noteworthy that the regulatory mechanisms of multi-organelle interactions, the relationship between the dynamic changes in mitochondrial morphology and function, and the specific role of the MMH network within this context require further in-depth investigation. These aspects will be a key focus of future research. Additionally, it must be clarified that there is an important gap between the proposed theoretical framework and its clinical application. Currently, the therapeutic theory that targets the MMH network is primarily based on research evidence from cellular and animal models, and its applicability and efficacy in human diseases still require extensive clinical research validation.
